# Attitudes of German GP trainees regarding add-on training programs differ if in office or hospital training phase

**DOI:** 10.1186/s12909-022-03273-2

**Published:** 2022-03-26

**Authors:** DMG Wild, K. Linden, T. Welchowski, D. Dehnen, B. Weltermann

**Affiliations:** 1 Institute of Family Medicine and General Practice, University Hospital Bonn, University of Bonn, Venusberg-Campus 1, D-53127 Bonn, Germany; 2 Institute of Medical Biometry, Informatics and Epidemiology, University Hospital Bonn, University of Bonn, Bonn, Germany; 3 Institute of Family Practice, Medical Faculty, University of Duisburg-Essen, Essen, Germany

**Keywords:** Primary care, Post-graduate medical education, Internship and residency, Curriculum

## Abstract

**Background:**

Many residents are exposed to negative attitudes towards primary care during hospital training. Attractive add-on training programs exist, but it is unclear whether these need to be tailored to the location of training (hospital vs. office). We report differences in learner attitudes from a large German add-on training program.

**Methods:**

Between 2017 and 2020, a regional network offered 31 quarterly seminars to primary care residents. The seminars addressed medical content, practice management and mentoring. We elicited participants’ satisfaction, perceived topic relevance, preferences for future seminars, work situation and employer support for participation. A proportionate odds model was used to assess predictors of ratings; results were stratified by training location (hospital vs. office).

**Results:**

Most respondents were female (380/575 = 70.0%), aged between 26 and 40 (80.8%), and had on average 3.54 ± 1.64 years of residency training. The majority (83.8%) was working in an office and full-time (63.0%). Overall evaluations were positive (very satisfactory 72.1%). Comparing residents in the hospital phase vs. the office phase, overall seminar ratings of the perceived impact on the motivation for primary care did not differ (*p* = 0.73 vs. 0.18, respectively). Hospital-based residents were less likely to rate the topics as relevant (39.4% vs. 55.7%, *p* = 0.02) and had different preferences for future seminar topics (top 3: palliative care, emergencies and chronic care vs. billing, disease management and practice finances for hospital and office phase, respectively).

**Conclusions:**

Keeping primary care residents motivated may require education tailored to training location. Our findings may be of interest to teachers, administrators and policymakers.

**Supplementary Information:**

The online version contains supplementary material available at 10.1186/s12909-022-03273-2.

## Background

Germany, like many other European countries, is struggling with a growing lack of primary care physicians. The reasons for this looming shortage are complex [1]. While many primary care physicians perceive their career choice as rewarding [2], many more medical students turn away from primary care due to a perceived lower status and unattractive work conditions [3]. This effect extends to residents as well: Alberti et al. report that British GP trainees perceive their field as having low status [4]. Previous studies have found that up to 50% of residents change career plans away from primary care [5]. Even among US-American residents in dedicated primary care programs, a significant percentage lose interest and do not end up in clinical primary care [6]. For all these reasons, it is all the more critical to motivate and retain those residents who are already in primary care training programs.

Maintaining residents’ motivation to pursue primary care is of particular importance in countries like Germany [7], where training comprises a hospital-based and an office-based phase [8]. The training sites are typically organised by the residents themselves. In the hospital phase, residents rotate in surgical or medical departments. In the office phase, residents are integrated in individual practices and receive most of their formal training through longitudinal add-on seminars. During hospital-based training, residents are often exposed to negative attitudes towards primary care [9]. Local attractive add-on training programs which include mentoring schemes have been well received [10–12], especially if they incorporate non-clinical aspects such as leadership and management [13, 14]. Such additional training programs are common internationally [15] and therefore relevant outside of Germany.

Increasing breadth of training has been found to improve GP recruitment and retention [16]. It is possible that hospital-based residents might benefit from support that is tailored to their situation and learning needs. To our knowledge, this has not been studied so far.

## Methods

### Program description

In 2017, a regional competency center for post-graduate education in family medicine was created through a joint effort of the regional medical chamber, the hospital association and the five university departments of family practice in the German North-Rhine region. The program is financed and set up within the framework provided by the German social law SGB V § 75a which was supplemented with a bundle of strategies to enhance the primary care workforce [17]. The centre is supported by the association of general practitioners and the regional chapter of the association of young family physicians (the association of young family physicians is a national network of residents and recent graduates of primary care training programs.) Throughout the year, the program offers voluntary seminar days which combine practical medical content and advice on practice management with mentoring, networking opportunities as well as career planning support. All seminars are held in small groups, are highly interactive and include training as well as networking opportunities with experienced family physicians. Between September 2017 and June 2020, a total of 31 seminar days with 8 teaching hours each were offered. Since April 2020, the program has been administered online only due to the COVID-19 pandemic.

In Germany, residents are not required to declare their specialty career goal at the start of their residency. Therefore, it is not clear at any given time how many future primary care residents are in hospital training (hospital time can be accredited towards both internal medicine or primary care certification), nor is participation in training seminars mandatory. Residency ends with an oral examination.

A few representative examples of the seminar days are shown below (see Table 1). The seminars end with a “next day pearl”, a message that trainees can share with their local supervisor the next day.

**Table 1:** Sample of seminar days

**A: Management of crises and emergencies in primary care**	
• Talk: Office structures that are helpful for emergency management	
• Talk: The ABCDEs of Advanced Life Support	
• Small-group activity with simulated patients: advanced life support	
• Mentoring activity: Small group discussions about legal rules for compulsory placement of patients in psychiatric units	
**B: Taboo topics in primary care:**	
• Talk: Diagnostics and therapeutics in addiction medicine	
• Plenary discussion: Addiction – an everyday topic?	
• Small-group activity: Sexuality and sexual disorders	
• Small-group activity: Gynaecology and urology for primary care physicians	

### Evaluation methods

From June 2017 to June 2020, all participants were surveyed after each seminar day about their satisfaction with the program, their preferences for future days and the perceived relevance of the program for their daily work. We also asked about their age group, work situation and how they were able to participate (paid leave, use of vacation days, etc.) The survey was developed by a group of experts based on previous work experience with the target group.

In regards to the seminar program, the survey asked about the satisfaction with each program part using a six-point Likert scale (featuring “very satisfied”, “satisfied”, “somewhat satisfied”, “somewhat dissatisfied”, “dissatisfied” and “very dissatisfied”). Respondents were asked to rate their impression of the competency gained, the opportunities for discussion, the content, didactics and the relevance for their daily practice.

Respondents also gave an overall rating for the day with six ratings corresponding to the country’s school grades: very good (1), good (2), satisfactory (3), adequate (4), poor (5) and very poor (6). Lastly, they were asked about their perceptions of the opportunities for networking, if the mentoring part of the seminar day strengthened their motivation to become a primary care physician, and which topics they were most interested in for future seminar offerings.

### Statistical analysis

Descriptive data were used for baseline characteristics. For each seminar day, we calculated the absolute and relative percentages of evaluations in each rating category (very satisfied, satisfied etc.). We also divided the program items into the categories “Therapy and diagnosis”, “Evidence-based medicine”, “Prevention”, “Practice management” and “Career planning”. For each category, we calculated the absolute and relative percentages of all evaluations in each category of the Likert scale. We also calculated the absolute and relative percentages of trainees who had taken paid leave for the seminar, comparing trainees who were in the hospital phase versus the office phase. In addition, we calculated a 6 × 6 table of the total score of the day and the perceived impact on the motivation to become a primary care physician.

To compare the overall ratings of the day with the perception that the mentoring part of the day increased the motivation to pursue primary care training, the marginal probability distributions were compared with a Monte Carlo permutation test (10^5^ simulations) regarding the sum of absolute differences of the relative category frequencies. Laplace smoothing was applied to better take into account low relative frequencies in categories. The top five wishes were compared between hospital and practice residents with a two-sample permutation test based on the Anderson-Darling test statistic (10^5^ simulations). Furthermore, we compared whether respondents differed with regard to receiving paid leave depending on whether they were hospital- based or office-based at the time of participating in the seminar through a contingency table with a Monte Carlo Chi^2^ independence test (10^5^ simulations). Comparisons between two relative frequencies were analysed by two-sample tests for equality of proportions using Yates’ continuity correction.

Lastly, we constructed a proportional odds model with inverse cumulative probabilities and logit-link [18] regarding 1) the overall rating of the seminar day and 2) the increase in the motivation to become a primary care physician. Most of the evaluations of these two questions were right-skewed, and negative assessments were rare. Therefore, the evaluation categories “very dissatisfied”, “dissatisfied”, “rather dissatisfied” and “rather satisfied” were aggregated to one category. Near-zero variability variables (population paediatric and geriatric, topic category evidence based-medicine) were excluded prior to modelling [19]. To reduce the number of variables, all questions regarding single seminars were converted to sum scores per seminar evaluation. Missing data were addressed by multiple imputation with chained equations [20]. All variables used in the proportional odds model were previously imputed. The number of imputations and iterations were both set to 25 using default imputation models of the R package mice according to the measurement scale of variables. The final proportional odds model coefficients were pooled [21]. The goodness of fit of the pooled proportional odds model was measured by average proportion of deviance explained across all imputed data sets [22]. Data were analysed using R 3.6.3 with packages VGAM_1.1–3, caret_6.0–86, ggplot2_3.3.0, lattice_0.20–41, MESS_0.5.6, car_3.0–8, carData_3.0–4, openxlsx_4.1.5 and mice_3.9.0.

## Results

### Respondents

Overall, we had 503 unique participants each year and 999 session participants, for an average of 2.0 seminar days attended per resident per year (median 1.7.) We obtained 575 evaluations (response rate 57.6%). Since the survey was anonymous, it was not possible to determine how many training sessions a particular resident attended. Therefore, the demographic data are participant-based. Baseline characteristics of our subjects are shown in Table 2. Our respondents were mostly female (70.0%), aged between 26 and 40 (80.8%), and had an average of 3.54 ± 1.64 years of residency training. The majority (83.8%) was currently in the office-based phase of their training, and most were working full-time (63.3%), with a sizable minority working part-time (32.6%). 81 surveys were returned by residents in the hospital phase, and 449 by residents in the office phase.

**Table 2:** Baseline characteristics of respondents (*n* = 575); Missing data as indicated

	N	%	Missing values (%)
**Age**			69 (12.0)
20–30	110	21.7	
31–35	191	37.8	
36–40	113	22.3	
> 40	92	18.2	
**Gender**			32 (5.6)
Male	163	30.0	
Female	380	70.0	
**Year of residency**:			76 (13.2)
Average +/− SD	3.54	1.6	
**Current training setting**			39 (6.8)
Hospital-based training	81	15.1	
Office-based training	449	83.8	
Other (e.g., parental leave)	6	1.1	
**Work situation**			25 (4.9)
Full-time	346	63.3	
Part-time	178	32.6	
On parental leave/not working	23	4.2	
**Employer support for seminar participation**			53 (9.2)
Paid leave	370	70.9	
Vacation day	48	9.2	
Free time compensation	16	3.1	
Other	88	16.9	

#### Program output

As of June 2020, 31 seminar days with a total of 170 topic items were offered (see Table 3). Most topics were related to diagnosis and therapy (73.1%) followed by practice management (23.5%). Other frequent topics included communication, self-reflection and self-care (17.1%) as well as career planning (11.2%) and prevention (8.2%).

**Table 3:** Overview of the program’s number of training days, training hours and seminar locations

	N	%
Total seminar days	31	
Total agenda items in those 31 days	170	
Agenda items in the categories		
Diagnosis and therapy	63	73.1%
Practice management	40	23.5%
Communication, reflection, self-care	29	17.1%
Career planning	19	11.2%
Prevention	14	8.2%
Evidence-based medicine	5	2.9%
Seminar days held in rural/underserved areas	4	13.0%
Average number of teaching hours	8/day	

#### Program evaluation

Overall evaluations of the seminar days were very positive (see Table 4). Most respondents rated the overall support as very satisfactory (59.7%) or satisfactory (36.2%). Ratings were similarly high in terms of their value for professional development (62.4% very satisfactory, 32.3% satisfactory) and the overall atmosphere (71.4% very satisfactory, 24.4% satisfactory). Overall, 59.7% rated the seminars as very satisfactory, 36.2% as satisfactory, 3.7% as rather satisfactory. Fewer respondents reported that the seminars strengthened their commitment to primary care (52.8% very satisfied, 34.7% satisfied). These differences did not reach statistical significance (*p* = 0.39).

**Table 4:** Respondents’ evaluations of the seminar days: overall rating and by category, *n* = 575

Category	Very satisfied (%)	Satisfied (%)	Rather satisfied (%)	Rather dissatisfied or dissatisfied (%)
Overall evaluation	320 (59.7)	194 (36.2)	20 (3.7)	2 (0.4)
Overall atmosphere	401 (71.4)	137 (24.4)	20 (3.6)	4 (0.7)
Value for professional development	349 (62.4)	180 (32.3)	25 (4.5)	5 (0.9)
Topic relevance	251 (53.3)	140 (29.9)	51 (10.9)	27 (6.0)
Strengthens motivation to be primary care physician	242 (52.8)	159 (34.7)	41 (9.0)	16 (3.5)
Opportunity for networking	404 (72.1)	135 (24.1)	20 (3.6)	1 (0.2)
**Agenda subcategories**				
Diagnosis and therapy	257 (59.5)	157 (36.3)	16 (3.7)	2 (0.5)
Prevention	50 (61.0)	27 (32.9)	4 (4.9)	1 (1.2)
Evidence-based medicine	17 (51.5)	14 (42.4)	1 (3.0)	1 (3.0)
Practice management	276 (57.6)	182 (38.0)	19 (4.0)	2 (0.4)
Communication, reflection, self-care	133 (61.9)	71 (33.0)	10 (4.7)	1 (0.5)
Career planning	179 (63.5)	94 (33.3)	9 (3.2)	0 (0)
**Seminar format**				
Plenary session	140 (65.1)	66 (30.7)	8 (3.7)	1 (0.5)
Small group discussion	272 (58.5)	172 (37.0)	19 (4.1)	2 (0.4)
Mentoring	247 (61.9)	141 (35.3)	11 (2.8)	0 (0)

Table 5 summarises the seminar evaluations stratified by training phase (hospital vs. office). Residents in the hospital phase were less likely to rate the topics as very relevant (39.4% vs. 55.7%, *p* = 0.02). There was no difference in the assessment of the seminars as regards strengthening the motivation to pursue primary care (43.8% vs. 53.9%, *p* = 0.18).

**Table 5:** Respondents’ evaluations of the seminar days by category, stratified by residents in different training settings (hospital phase versus office phase), missing data not shown; significant differences bolded

Category	Participants in hospital phase (***n*** = 81)				Participants in office phase (***n*** = 449)			
	Very satisfied (%)	Satisfied (%)	Rather satisfied (%)	Rather dissatisfied or worse (%)	Very satisfied (%)	Satisfied (%)	Rather satisfied (%)	Rather dissatisfied or worse (%)
Overall evaluation	46 (61.3)	28 (37.3)	1 (1.3)	0 (0)	251 (59.3)	152 (35.9)	18 (4.3)	2 (0.5)
Overall atmosphere	60 (74.1)	21 (25.9)	0 (0)	0 (0)	313 (70.8)	108 (24.4)	19 (4.3)	2 (0.5)
Value for professional development	49 (61.3)	28 (35.0)	2 (2.5)	1 (1.3)	275 (62.4)	142 (32.2)	21 (4.8)	3 (0.7)
**Topic relevance**	**26 (39.4)**	**28 (42.4)**	**9 (13.6)**	**3 (4.6)**	**206 (55.7)**	**101 (27.3)**	**40 (10.8)**	**23 (6.2)**
Strengthens motivation to be primary care physician	28 (43.8)	24 (37.5)	10 (15.6)	2 (3.1)	196 (53.9)	125 (34.3)	30 (8.2)	13 (3.6)
Opportunity for networking	61 (76.3)	17 (21.3)	2 (2.5)	0 (0)	313 (71.1)	113 (25.7)	14 (3.2)	0 (0)
**Agenda subcategory**								
Diagnosis and therapy	36 (59.0)	24 (39.3)	1 (1.6)	0 (0)	204 (59.1)	125 (36.2)	14 (4.1)	2 (0.6)
Prevention	6 (54.6)	4 (36.4)	1 (9.1)	0 (0)	39 (60.0)	22 (33.9)	3 (4.6)	1 (1.5)
Evidence-based medicine	2 (66.7)	1 (33.3)	0 (0)	0 (0)	13 (48.2)	12 (44.5)	1 (3.7)	1 (3.7)
Practice management	36 (56.3)	27 (42.2)	1 (1.6)	0 (0)	219 (57.8)	141 (37.2)	17 (4.5)	2 (0.5)
Communication, reflection, self-care	20 (66.7)	9 (30.0)	1 (3.3)	0 (0)	100 (60.6)	55 (33.3)	9 (5.5)	1 (0.6)
Career planning	21 (67.7)	10 (32.3)	0 (0)	0 (0)	148 (63.0)	78 (33.2)	9 (3.8)	0 (0)
**Teaching format**								
Plenary session	24 (75.0)	7 (21.9)	1 (3.1)	0 (0)	101 (63.12%)	52 (32.5)	6 (3.8)	1 (0.6)
Small group discussion	41 (60.3)	26 (38.2)	1 (1.5)	0 (0)	209 (57.9)	133 (36.8)	17 (4.7)	2 (0.6)
Mentoring	37 (62.7)	22 (37.3)	0 (0)	0 (0)	194 (62.4)	107 (34.4)	10 (3.2)	0 (0)

**Fig. 1:**
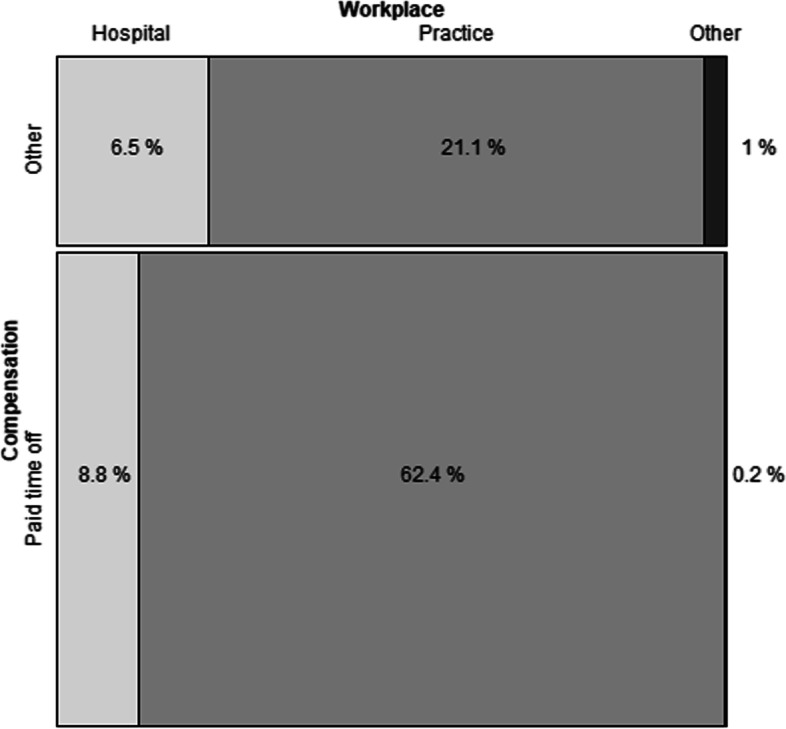
Employer support for seminar attendance by training phase

Residents in the hospital phase differed from those in the office phase in respect to the topics they found most interesting (see Table 6). Office-based residents were more interested in practice management topics than hospital-based residents. The only topic that was in both groups’ top 5 was the category clinical examination techniques. Employer support for attending the seminars differed significantly (see Fig. 1): While most residents in the practice phase received time off (74.7%), only slightly more than half (57.7%) of the trainees in the hospital phase received this benefit (*p* < 0.01).

**Table 6:** Respondents’ top 10 preferences for future seminar topics and employer support for participation, stratified by training site (*n* = 575); multiple answers were possible

Residents in hospital phase	Residents in office phase
Topic (%)	Topic (%)
Palliative care (41.3%)	Billing (47.8)
Emergencies (40.0)	Disease management programs (44.4)
Chronic care (38.3)	Practice finances (37.3)
Clinical examination techniques (38.3)	Practice management (35.4)
Psychosomatic medicine (35.8)	Clinical examination techniques (34.6)
Disease management programs (34.2)	Contracts (33.8)
Practice management (33.8)	Palliative care (33.3)
Communication with challenging patients (32.9)	Emergencies (32.0)
Contracts (31.3)	Establishing a practice (30.2)
Prevention (31.3)	Geriatrics (27.9)

#### Proportional odds model

In the proportional odds model, neither age group, gender, training year, training content, nor training phase had an impact on the overall rating. However, the number of training hours per seminar and the positive ratings of individual components of the program did have an impact (for training hours odds ratio (OR) 2.08, confidence interval (CI) 1.15–3.77, *p* = 0.02; for seminar rating items 5–9 OR 1.2416, CI 1.14–1.36, *p* < 0.01; seminar rating items 10–14 OR 1.1868, CI 1.08–1.31, *p* < 0.01; details see Additional file 1). There was a trend towards higher ratings for seminars that included mentoring; however, this factor did not reach statistical significance (OR 3.37, CI 0.65–17.61, *p* = 0.15). The goodness of fit of the pooled proportional odds model, as measured by average proportion of deviance explained across all imputed data set, was 11.3% (for details, see Additional file 1).

Several factors predicted residents’ perception of the mentoring part of the program to strengthen their motivation to pursue primary care, namely being in the office-based training phase (OR 1.88, CI 1.06–3.35; *p* = 0.03), as well as programs featuring topics within the categories prevention (OR 1.43, CI 1.14–1.79, *p* < 0.01) or practice management (OR 1.45; CI 1.08–1.93, *p* = 0.01). The goodness of fit of the pooled proportional odds model, as measured by average proportion of deviance explained across all imputed data set, was 28%.

## Discussion

We report evaluations of a longitudinal add-on training program and differences in training evaluations depending on the training phase of residents. We found the program to be overall well received, with positive ratings particularly for the topics prevention and practice management. Our findings supplement the existing literature in important ways. Broerman et al. studied preferences of physicians in training for mentoring programs [23] and their satisfaction with a mentoring program in a small sample (*n* = 21) [24]. Our results confirm the value of mentoring in a much larger sample. Hoffman, Flum and Steinhäuser described results of email requests for mentoring, which also substantiate the important role of information around practice management and career planning for residents [25].

Residents in the hospital phase differed from those in the office phase in terms of their training preferences, preferred topics and training needs. Stanley et al. hypothesised that the decreasing interest in primary care in their sample was caused by the inpatient focus of residency training and suboptimal experiences in the ambulatory clinic, but they did not evaluate the role of dedicated training sessions [6]. Improving primary care teaching of residents in the hospital phase may also have an impact on the general culture of academic medical centres, which are often described as having a negative attitude towards primary care [3, 26, 27], and may attract other residents to primary care. This would be a significant strategy, since many countries like Germany are facing a surplus of residents aiming to pursue specialist care but a lack of primary care physicians [28, 29]. This strategy may also have an impact on medical students’ career choices, which are impacted by the perceived status and intellectual challenges of various career paths [29].

While three quarters of the residents in the practice phase received paid leave, this was true for only slightly more than half of the trainees in the hospital phase. Since the salary of primary care residents is heavily subsidised by the statutory and private health insurances, these findings are surprising. This effect may have been due to the workload in the hospital setting. It is also possible that residents in the hospital phase attended hospital-based teaching seminars instead.

Ultimately, the value of a training program lies in its ability to increase the supply of motivated and competent primary care physicians. Our results suggest that such programs should be tailored to the different phases of training. While our findings must be regarded as preliminary, and while many other external factors impact motivation [30], this question merits further research.

### Strengths and limitations

We report on a large number of training seminars from one of the largest add-on training areas in the country. Since seminar attendance is voluntary, participants seem to find our programme valuable, which is reflected in our ratings. To our knowledge, our study is the first to stratify by training phase and to perform a sophisticated statistical analysis of the components of the training program and their impact on overall ratings. However, the study should also be interpreted in light of its limitations. We surveyed a sample of regional trainees, which may not be representative of all German trainees. We were not able to trace individual residents and can therefore draw no conclusions regarding the development of interests in different topics over the course of the residency. This lack of traceability may have led to some residents participating in the survey in both the hospital and the office phase. In addition, we were unable to calculate attendance rates for our seminars based on the training phase, since residents are not required to declare their specialty and can also change their intended specialisation during residency. Course participation is not mandatory, which may have skewed participants’ ratings of the courses towards the positive. The statistical model assumes that all odds ratios across categories do not vary, but the sample size was too small to substantiate this assumption.

## Conclusion

In summary, longitudinal supplemental training seems to be a promising method to increase the supply of motivated primary care physicians. The content should be tailored to the actual training phase of the residents in order to maximise motivation. We hope that our results will be helpful to other programs and countries.

## Supplementary Information

**Additional file 1.**

## Data Availability

The data used in this study is available from the corresponding author on reasonable request.
