# Supplementary material for: Attitudes of German GP trainees regarding add-on training programs differ if in office or hospital training phase
Source: BMC Med Educ. 2022 Mar 26;22:205. doi: 10.1186/s12909-022-03273-2 (PMC8959794; doi:10.1186/s12909-022-03273-2)
Supplement: Supplementary file 1 — Additional file 1. [file 12909_2022_3273_MOESM1_ESM.pdf]

Appendix: Supplementary material

Table S1: Proportiononal odds model estimating probability of observing at least a given overall rating category; LL=lower limit, CI=confidence interval, UL=upper limit, e=exponent, p-values < 0.05 in bold

**Description**	**Odds ratio**	**LL CI**	**UL CI**	**p-Value**
Teaching format mentoring	3,3743	0,6465	17,6127	1.50e-01
Part of training association: Free time compensation	2,4744	0,5621	10,8921	2.32e-01
Working situation: currently not working	2,2676	0,1189	43,2462	5.87e-01
**Training hours**	**2,0842**	**1,1529**	**3,7678**	**1.54e-02**
Workshop place 1	1,5896	0,3231	7,8199	5.69e-01
Working place: Other	1,451	0,0859	24,5169	7.97e-01
**Complete Evaluation seminar day sum scores items 1-3**	**1,4152**	**1,224**	**1,6364**	**3.29e-06**
Part of training association: Other	1,3354	0,6671	2,6733	4.15e-01
**First seminar sum scores items 5-9**	**1,2416**	**1,1377**	**1,355**	**1.65e-06**
Professional development: Yes	1,2239	0,6988	2,1436	4.81e-01
Part of training association: Vacation day	1,2104	0,5151	2,8444	6.62e-01
Teaching format talk	1,2005	0,4807	2,9983	6.96e-01
**Second seminar sum scores items 10-14**	**1,1868**	**1,0775**	**1,3071**	**5.57e-04**
Teaching format small group training	1,1746	0,5238	2,6337	6.96e-01
Work place: office	1,1326	0,5484	2,3388	7.37e-01
Workshop place 2	1,0757	0,2887	4,0083	9.13e-01
Fifth seminar sum scores items 25-29	1,0619	0,9875	1,1419	1.09e-01
Topic category prevention	1,0604	0,4932	2,2799	8.81e-01
Fourth seminar sum scores items 20-24	1,031	0,9693	1,0967	3.34e-01
Age	1,0197	0,9742	1,0673	4.03e-01
Third seminar sum scores items 15-19	1,0131	0,9322	1,1012	7.59e-01
Year of residency	0,9834	0,8397	1,1516	8.35e-01
Topic category wishes sum scores	0,9718	0,9012	1,048	4.58e-01
Teaching format plenary discussion	0,9546	0,3623	2,5154	9.25e-01
Patient population general medicine	0,9505	0,7432	1,2157	6.86e-01
Topic category diagnosis + therapy	0,8471	0,4473	1,6043	6.11e-01
Working situation: part time	0,7894	0,1724	3,6145	7.61e-01
Topic category communication/reflection/selfcare	0,7553	0,4007	1,4236	3.86e-01
Workshop place 3	0,7449	0,2145	2,5872	6.43e-01
Working situation_full time	0,7388	0,155	3,5207	7.04e-01
Gender: Male	0,7111	0,4205	1,2024	2.04e-01
Workshop place 4	0,6932	0,1323	3,6324	6.65e-01
Topic category practice management	0,643	0,303	1,3646	2.50e-01
Topic category professional development	0,5961	0,1916	1,8541	3.72e-01

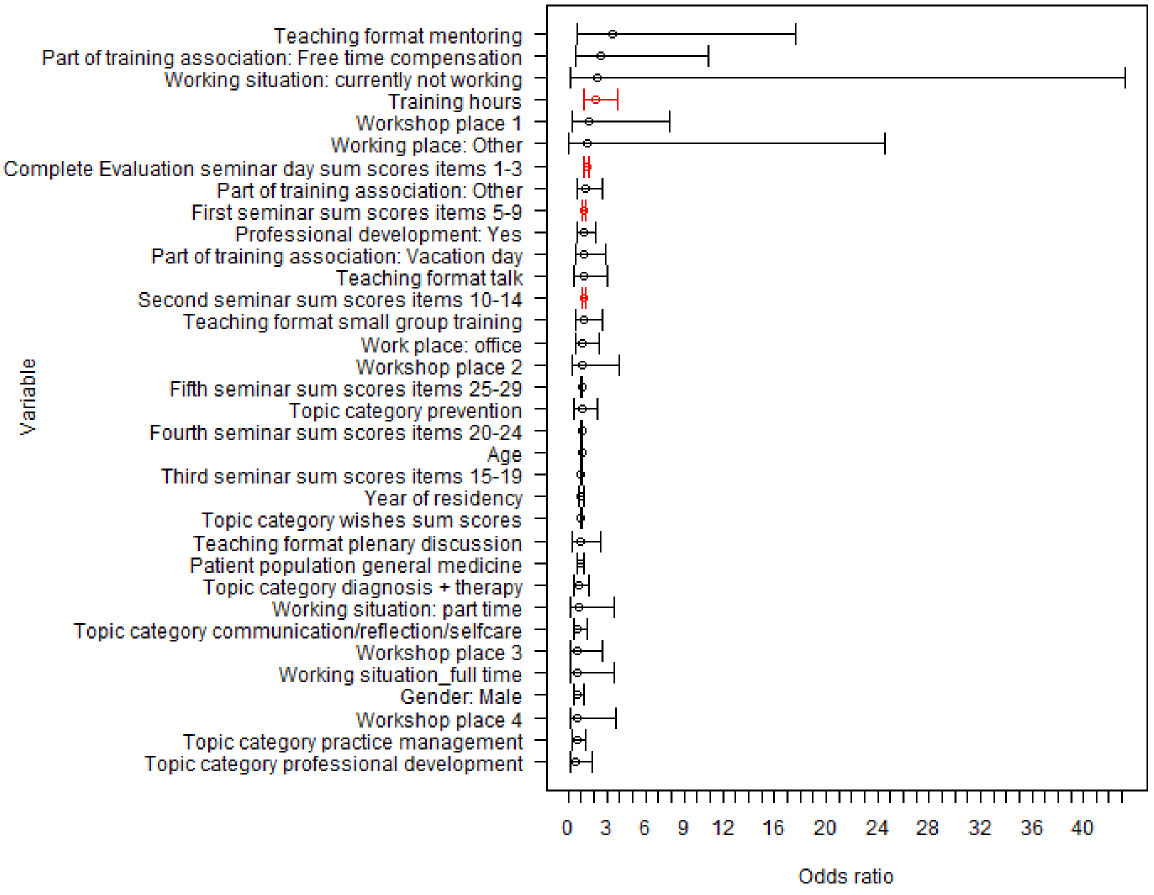

Supplementary Figure 1: Proportional odds model estimating probability of observing at least a given overall rating category, significant variables in red

Table S2: Proportiononal odds model estimating probability of observing at least a given category in question “mentoring part of the day strengthens my motivation to go into primary care”; LL=lower limit, CI=confidence interval, UL=upper limit, e=exponent, p-values < 0.05 in bold

**Description**	**Odds ratio**	**Lower CI**	**Upper CI**	**p-Value**
**Work place: Office**	**1,8791**	**1,0558**	**3,3445**	**3.29e-02**
Part of training association_Other	1,4816	0,7887	2,7832	2.23e-01
**Topic category: practice management**	**1,446**	**1,0806**	**1,9348**	**1.33e-02**
**Topic category: prevention**	**1,4279**	**1,1415**	**1,7861**	**2.05e-03**
Training hours	1,3005	0,8765	1,9296	1.93e-01
Professional development: Yes	1,2118	0,8085	1,8163	3.53e-01
Complete Evaluation seminar day sum scores items 1-3	1,14	0,9883	1,3151	7.42e-02
**First seminar sum scores items 5-9**	**1,1386**	**1,0581**	**1,2252**	**5.80e-04**
Topic category: communication/reflection/selfcare	1,1224	0,8813	1,4294	3.50e-01
Topic category: professional development	1,1206	0,7271	1,7269	6.06e-01
Topic category: diagnosis & therapy	1,0628	0,8605	1,3126	5.72e-01
Second seminar sum scores items 10-14	1,0557	0,9633	1,157	2.48e-01
Fourth seminar sum scores items 20-24	1,0492	0,9935	1,1079	8.64e-02
Third seminar sum scores items 15-19	1,0181	0,9437	1,0983	6.44e-01
Age	1,0077	0,97	1,047	6.93e-01
Part of training association; Free time compensation	0,9853	0,305	3,1829	9.80e-01
Topic category wishes sum scores	0,9681	0,9047	1,0359	3.49e-01
Year of residency	0,9426	0,8281	1,0731	3.73e-01
Part of training association: Vacation day	0,9371	0,4647	1,8895	8.56e-01
**Patient population general medicine**	**0,82**	**0,6894**	**0,9753**	**2.61e-02**
Working situation_part time	0,8171	0,2088	3,1976	7.72e-01
Working situation_full time	0,7686	0,1929	3,0615	7.09e-01
Gender_Male	0,7226	0,4562	1,1444	1.67e-01

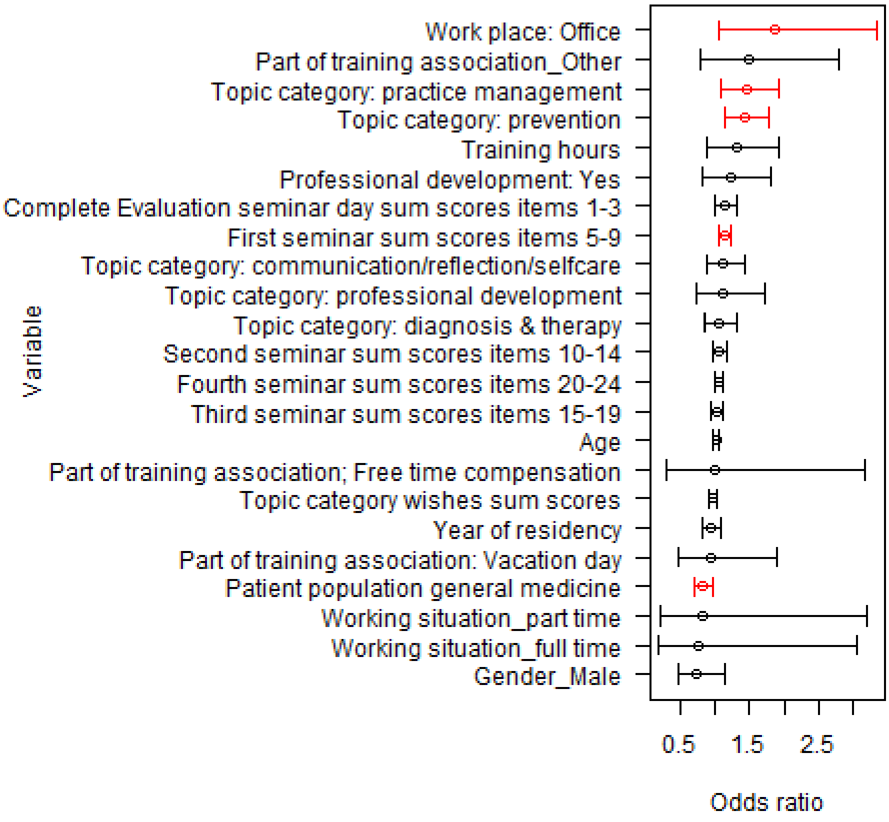

Supplementary Figure 2: Proportional odds model estimating probability of observing at least a given category in question mentoring part strengthens motivation to go into primary care; significant variables in red
